# Prevalence of extended-spectrum β-lactamase-producing *Enterobacterales* in edible ice in Thailand

**DOI:** 10.1093/inthealth/ihae050

**Published:** 2024-07-29

**Authors:** Premjit Amornchai, Vanaporn Wuthiekanun, Sayan Langla, Gumphol Wongsuvan, Panatda Aramrueang, Nicholas P J Day, Direk Limmathurotsakul

**Affiliations:** Mahidol-Oxford Tropical Medicine Research Unit, Faculty of Tropical Medicine, Mahidol University, Bangkok 10400, Thailand; Mahidol-Oxford Tropical Medicine Research Unit, Faculty of Tropical Medicine, Mahidol University, Bangkok 10400, Thailand; Mahidol-Oxford Tropical Medicine Research Unit, Faculty of Tropical Medicine, Mahidol University, Bangkok 10400, Thailand; Mahidol-Oxford Tropical Medicine Research Unit, Faculty of Tropical Medicine, Mahidol University, Bangkok 10400, Thailand; Clinical Microbiology Department, Sunpasitthiprasong Hospital, Ubon Ratchathani 34000, Thailand; Mahidol-Oxford Tropical Medicine Research Unit, Faculty of Tropical Medicine, Mahidol University, Bangkok 10400, Thailand; Centre for Tropical Medicine and Global Health, Nuffield Department of Medicine, University of Oxford, OX3 7FZ, UK; Mahidol-Oxford Tropical Medicine Research Unit, Faculty of Tropical Medicine, Mahidol University, Bangkok 10400, Thailand; Centre for Tropical Medicine and Global Health, Nuffield Department of Medicine, University of Oxford, OX3 7FZ, UK; Department of Tropical Hygiene, Faculty of Tropical Medicine, Mahidol University, Bangkok 10400, Thailand

**Keywords:** antimicrobial resistance, drug resistant, edible ice, ESBL-producing *Enterobacterales*, Thailand

## Abstract

**Background:**

The presence of antimicrobial-resistant (AMR) bacteria in edible ice in tropical countries is largely unknown.

**Methods:**

We evaluate the presence of extended-spectrum β-lactamase (ESBL)-producing *Enterobacterales* in 100 edible ice samples from drink carts in 20 markets in four provinces (five markets/province) in Thailand. Ten samples of commercially sold edible ice in sealed packages were tested as controls.

**Results:**

Of 100 samples, 29 (29%) were culture positive for ESBL-producing *Enterobacterales*, with a median quantitative count of 2 colony-forming units (CFU)/100 mL (range, 1 to 40 CFU/100 mL). All control samples were culture negative for ESBL-producing *Enterobacterales*.

**Conclusions:**

AMR bacteria is commonly found in edible ice from drink carts.

## Introduction

Travel-acquired extended-spectrum β-lactamase (ESBL) colonization is common among those who travel to Africa, South Asia and Southeast Asia.^1^ In Thailand, ESBL-producing *Escherichia coli* and *Klebsiella pneumoniae* are major causes of community-acquired antimicrobial-resistant (AMR) bacterial infection and are commonly found in ground water, farm wastewater and hospital wastewater.^2^

It is generally recommended for travelers visiting Thailand to drink bottled water.^3^ All bottled water with Thai Industrial Standard (TIS) labels should have no *E. coli* and other pathogenic bacteria in the water, and have coliform bacteria <2.2 colony-forming units (CFU)/100 mL.^4^

However, the risk of ESBL colonization through consumption of edible ice has not been fully investigated.^1^ In tropical countries, edible ice is commonly consumed together with bottled water and soft drinks sold at drink carts and in coffee shops and restaurants, making it a potentially significant source of bacterial transmission. The current study aims to assess the prevalence of ESBL-producing *Enterobacterales* in edible ice in Thailand.

## Methods

We bought 100 edible ice samples from 20 markets (5 samples/market) in four provinces (Bangkok, Chiang Mai, Chonburi and Phuket; 5 markets/province) in Thailand. The markets were selected because they are famous among tourists. In each market, five drink carts were selected by convenience sampling. At each drink cart, we bought a full cup of ice (about 300 mL size) and immediately transferred it into a sterile plastic bag. Another 10 ice samples in sealed plastic packages with a label from the Thai Food and Drug Administration (Thai FDA) were bought from convenience stores (2–3 samples/province) and used as controls. All samples were stored in a cold chain container during transportation and processed in a laboratory in Bangkok within 48 h of collection.

In the laboratory, each ice specimen was melted at room temperature. The melted ice samples of 1, 10 and 100 mL were filtered through 0.45 µM of nitrocellulose membrane filters (Merck Millipore, Darmstadt, Germany) using a 3-branch manifold system (Sartorius, Bohemia, NY, USA) at room temperature. Each filter was placed on Brilliance ESBL (BE) agar (Oxoid, Basingstoke, UK) plates (i.e. three plates per sample) for the bacterial culture and quantitative counts. The agar plates were incubated at 37°C for 48 h.

After incubation, the colonies that grew on the plates with either a blue or pink color, indicating the presence of ESBL-producing *E. coli*, or a green color, indicating the presence of ESBL-producing *Klebsiella, Enterobacter, Serratia* and *Citrobacter* groups, were counted and collected for species identification. The collected isolates were then tested for ESBL production using the Clinical and Laboratory Standards Institute (CLSI)-combined disk diffusion methods and for species identification using an automated VITEK 2 XL system 9.01 (bioMérieux, Durham, NC, USA).

## Results

A total of 159 organisms were isolated from 29 ice samples on BE agar plates. Eight isolates were identified as non-ESBL producers (*Acinetobacter baumannii* [n=6]*, Acinetobacter pittii* [n=1] and *Leclercia adecarboxylata* [n=1]) and were excluded from further analysis.

**Figure 1.**
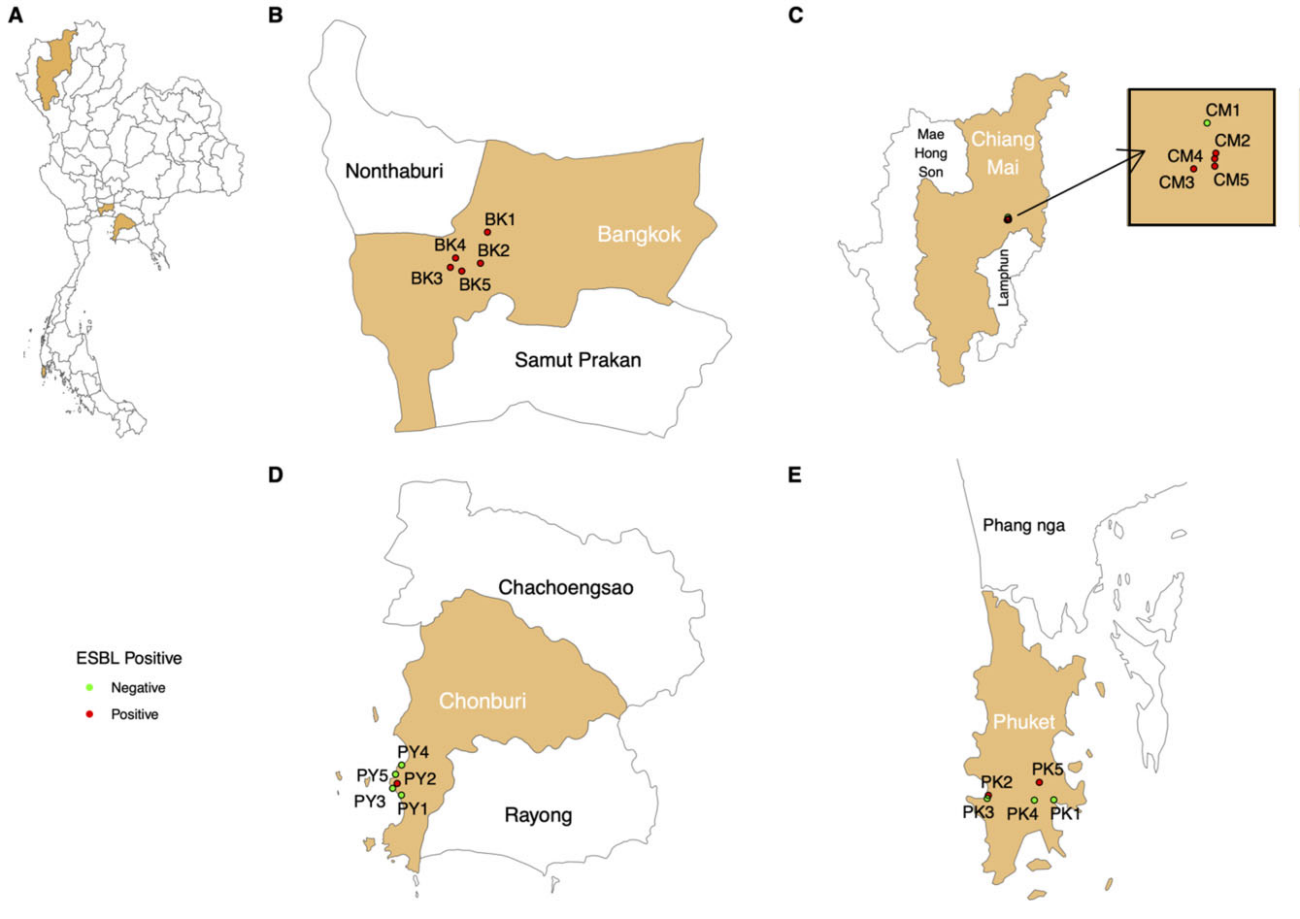
Study locations. (A) Map of Thailand showing four provinces where edible ice samples were collected; including Bangkok (B), Chiang Mai (C), Chonburi (D) and Phuket (E). Red dots represent locations (markets) with an edible ice sample culture positive ESBL-producing *Enterobacterales* and green dots represent locations (markets) with all five edible ice samples culture negative for ESBL-producing *Enterobacterales.*

Of 100 edible ice samples, 29 (29%) were culture positive for ESBL-producing *Enterobacterales*. The highest prevalence was found in Bangkok (72%; 18/25), followed by Chiang Mai (28%; 7/25), Phuket (12%; 3/25) and Chonburi (4%; 1/25) (Figure 1). The viable count of ESBL-producing *Enterobacterales* in the edible ice sample ranged from 1 to 40 CFU/100 mL, with a median count of 2 CFU/100 mL. All 10 control samples were culture negative for ESBL producers.

Twelve bacterial species were identified among 151 ESBL-producing *Enterobacterales* isolates. *Klebsiella pneumoniae* was the most common organism (n=69, 46%), followed by *E. coli* (n=42, 28%), *Raoultella planticola* (n=11, 7%), *Citrobacter freundii* (n=7, 5%), *Serratia odorrifera* (n=7, 5%), *Enterobacter cloacae* (n=4, 3%), *L. adecarboxylata* (n=3, 2%), *Serratia fonticola* (n=3, 2%), *Citrobacter braakii* (n=2, 1%), *Escherichia fergusonii* (n=1, 0.7%), *Pantoea* spp. (n=1, 0.7%) and *Raoultella ornithinolytica* (n=1, 0.7%). Overall, none were resistant to amikacin, ertapenem and meropenem, one was resistant to piperacillin-tazobactam (one *K. pneumoniae*), three were resistant to imipenem (three *S. fonticola*) and eight were resistant to cefepime (five *C. freundii*, two *E. coli* and one *E. fergusonii*).

## Discussion

Our finding that edible ice sold at drink carts in Thailand is commonly contaminated with ESBL-producing bacteria is a public health concern. This could be the source of ESBL colonization and potential health risks. Consuming ESBL-producing *E. coli* and *K. pneumoniae* could cause acute diarrhea and lead to *K. pneumoniae* infections, respectively.^5^ Outbreaks of infections caused by contaminated edible ice are occasionally reported in low- and middle-income countries (LMICs). Nonetheless, it is also possible that a proportion of patients with AMR infections in LMICs were caused by consuming contaminated food or drinks unknowingly.

Our findings may be applicable to many LMICs. We recommend that local people and travelers visiting LMICs avoid consuming edible ice at drink carts and in coffee shops and restaurants. If needed, they should only consume commercially sold edible ice in sealed packages approved by the national authorities. National authorities in tropical LMICs, especially Thailand, must enhance the quality standards of edible ice at drink carts and in coffee shops and restaurants to further promote street food tourism.

Our study has some limitations. First, we did not assess whether the source of the contamination was from the water, the ice factory, the transportation or the drink carts. Second, genetic evaluation is not included in this study. Third, the ESBL agars have low specificity when samples are mixed with other contaminants. Fourth, our study sample size might not be large enough to provide conclusive evidence. Therefore, our study may have overestimated or underestimated the presence and quantitative count of ESBL-producing bacteria in edible ice.

## Supplementary Material

ihae050_Supplemental_File

## Data Availability

The data that support this study are available at https://doi.org/10.6084/m9.figshare.23617737.
