# Supplementary material for: Prevalence of extended-spectrum β-lactamase-producing Enterobacterales in edible ice in Thailand
Source: Int Health. 2024 Jul 29;17(2):232–4. doi: 10.1093/inthealth/ihae050 (PMC11879576; doi:10.1093/inthealth/ihae050)
Supplement: ihae050_Supplemental_File [file ihae050_supplemental_file.docx]

**Appendix: Prevalence of Extended-spectrum β-lactamase (ESBL)-producing *Enterobacterales* in edible ice in Thailand**

# Additional methods

We bought 100 edible ice samples from 100 drink carts in 20 markets (five drink carts/market) in four provinces (Bangkok, Chiang Mai, Chonburi and Phuket, with five markets/province) in Thailand. We selected markets which were famous among tourists. At each drink cart, we bought a full cup of ice (about 300 mL). Characters of edible ice samples were categorized into crushed ice, tube ices and cube ices (Figure S1).

The color colonies from all inoculated ESBL agar plates, which grew as either blue or pink (indicative of ESBL-producing *Escherichia coli*) or as green (indicative of ESBL-producing *Klebsiella*, *Enterobacter*, *Serratia* and *Citrobacter* groups), were purified, identified and counted. Briefly, all color colonies were selected for subculture on MacConkey agar and Columbia agar. Oxidase testing was performed to recruit only oxidase-negative bacteria, and the isolated colonies were preserved in bacterial preservative broth for further testing, including bacterial identification, antimicrobial susceptibility testing, and ESBL production confirmation. If each color colony was mixed with other contaminants (colorless colonies) on the cultured plate, it was subjected to multiple rounds of subculture until a pure colony could be isolated. The bacterial count in CFU/100mL was determined based on the number of color colonies confirmed as ESBL producers.

The antimicrobial susceptibility testing was performed using automated VITEK 2 XL system version 9.01 (bioMérieux, Durham, NC, USA). The organisms were tested with 11 antimicrobial agents; including amikacin (AK), amoxicillin-clavulanic acid (AMC), cefepime (FEP), cefoperazone-sulbactam (SCF), ceftazidime (CAZ), ceftriaxone (CRO), ciprofloxacin (CIP), ertapenem (ETP), imipenem (IPM), meropenem (MEM), piperacillin-tazobactam (TZP). The minimum inhibitory concentration (MIC) value of each antimicrobial agent was interpreted as susceptible (S), intermediate (I), susceptible dose dependent (SDD) and resistance (R) as CLSI guidelines.^1^

We compared category variables using Chi-square test or Fisher’s exact test as appropriate. Data analysis was performed using GraphPad Prism version 10.0.3.

# Additional results

There were 333 color colonies that were suspected as ESBL producing *Enterobacterales* on Brilliance ESBL agar plates. A total of 159 color colonies were purified and isolated from 29 samples whereas the remaining of 174 colonies were lost during the purification due to the overgrowth of other contaminants (colorless colonies) on the same agar plate. A total of 151 organisms from 29 samples were verified as ESBL producers (Table S1). Eight isolates from four samples were verified as non-ESBL producers (*Acinetobacter baumannii* [n=6]*,* *Acinetobacter pittii* [n=1], and *Leclercia adecarboxylata* [n=1]) were excluded from further analysis.

Of 100 edible ice samples collected, 51 (51%) were classified as tube ice, 42 (42%) were crushed ice and 7 (7%) were cube ice. The prevalence of ESBL-producing *Enterobacterales* was highest in crushed ice (36%, 15/42 samples), followed by tube ice (25%, 13/51 samples) and cube ice (14%, 1/7 samples). However, the difference was not statistically significant (p=0.38).

The resistance rates for 11 antimicrobial agents were varied among ESBL-producing *Enterobacterales* organisms (Table S2).

**Supplementary Tables**

**Table S1. ESBL producing *Enterobacterales* isolated from edible ice in each province**

| Provinces | Markets | Types of market | No. of sample | No. of sample culture positive for non  ESBL-producing *Enterobacterales* | No. of sample culture positive for  ESBL-producing *Enterobacterales* | Percentage of samples culture-positive for ESBL-producing *Enterobacterales* in the province |
| --- | --- | --- | --- | --- | --- | --- |
| Bangkok | BK1 | Day market | 5 | 2 | 5 |  |
| (BK) | BK2 | Day market | 5 | 0 | 3 |  |
|  | BK3 | Day market | 5 | 0 | 2 | 72% (18/25) |
|  | BK4 | Day market | 5 | 0 | 4 |  |
|  | BK5 | Day market | 5 | 1 | 4 |  |
| Chiang Mai | CM1 | Day market | 5 | 0 | 0 |  |
| (CM) | CM2 | Day market | 5 | 0 | 3 |  |
|  | CM3 | Night market | 5 | 1 | 1 | 28% (7/25) |
|  | CM4 | Night market | 5 | 0 | 1 |  |
|  | CM5 | Night market | 5 | 0 | 2 |  |
| Chonburi | PY1 | Day market | 5 | 0 | 0 |  |
| (PY) | PY2 | Night market | 5 | 0 | 1 |  |
|  | PY3 | Night market | 5 | 0 | 0 | 4% (1/25) |
|  | PY4 | Day market | 5 | 0 | 0 |  |
|  | PY5 | Day market | 5 | 0 | 0 |  |
| Phuket | PK1 | Day market | 5 | 0 | 0 |  |
| (PK) | PK2 | Night market | 5 | 0 | 2 |  |
|  | PK3 | Night market | 5 | 0 | 0 | 12% (3/25) |
|  | PK4 | Night market | 5 | 0 | 0 |  |
|  | PK5 | Night market | 5 | 1 | 1 |  |
| Total |  |  | 100 | 4 | 29 | 29% (29/100) |

**Table S2. Resistance rates of ESBL-producing *Enterobacterales* isolated from the edible ice samples (n=151)**

|  | No. of tested isolates | Percentage of resistances (%) | | | | | | | | | | |
| --- | --- | --- | --- | --- | --- | --- | --- | --- | --- | --- | --- | --- |
| Organisms |  | AK | AMC | FEP | SCF | CAZ | CRO | CIP | ETP | IPM | MEM | TZP |
| *Citrobacter braakii* | 2 | 0 | 100 | 0 | 0 | 0 | 100 | 100 | 0 | 0 | 0 | 0 |
| *Citrobacter freundii* | 7 | 0 | 14 | 71 | 0 | 0 | 100 | 14 | 0 | 0 | 0 | 0 |
| *Enterobacter cloacae* | 4 | 0 | 50 | 0 | 0 | 50 | 100 | 0 | 0 | 0 | 0 | 0 |
| *Escherichia fergusonii* | 1 | 0 | 0 | 100 | 0 | 100 | 100 | 0 | 0 | 0 | 0 | 0 |
| *Escherichia coli* | 42 | 0 | 2 | 5 | 0 | 26 | 100 | 62 | 0 | 0 | 0 | 0 |
| *Klebsiella pneumoniae* | 69 | 0 | 3 | 0 | 0 | 25 | 100 | 67 | 0 | 0 | 0 | 1 |
| *Leclercia decarboxylata* | 3 | 0 | 0 | 0 | 0 | 0 | 100 | 33 | 0 | 0 | 0 | 0 |
| *Pantoea spp.* | 1 | 0 | 0 | 0 | 100 | 0 | 100 | 100 | 0 | 0 | 0 | 0 |
| *Raoultella ornithinolytica* | 1 | 0 | 0 | 0 | 0 | 0 | 100 | 100 | 0 | 0 | 0 | 0 |
| *Raoultella planticola* | 11 | 0 | 0 | 0 | 0 | 0 | 100 | 100 | 0 | 0 | 0 | 0 |
| *Serratia fonticola* | 3 | 0 | 100 | 0 | 0 | 0 | 100 | 0 | 0 | 100 | 0 | 0 |
| *Serratia odorrifera* | 7 | 0 | 0 | 0 | 0 | 0 | 100 | 0 | 0 | 0 | 0 | 0 |
| Total | 151 | 0 | 7 | 5 | 1 | 21 | 100 | 59 | 0 | 2 | 0 | 1 |

**Footnote of Table S2.** A total of 11 antimicrobials were tested, AK; Amikacin, AMC; Amoxicillin-clavulanic acid, FEP; Cefepime, SCF; Cefoperazone-Sulbactam, CAZ; Ceftazidime, CRO; Ceftriaxone, CIP; Ciprofloxacin, ETP; Ertapenem, IPM; Imipenem, MEM; Meropenem, TZP; Piperacillin-tazobactam.

## Supplementary Figure

**Figure S1**. **Characteristic of edible ice samples.** Crushed ice (A), tube ice (B) and cube ice (C).


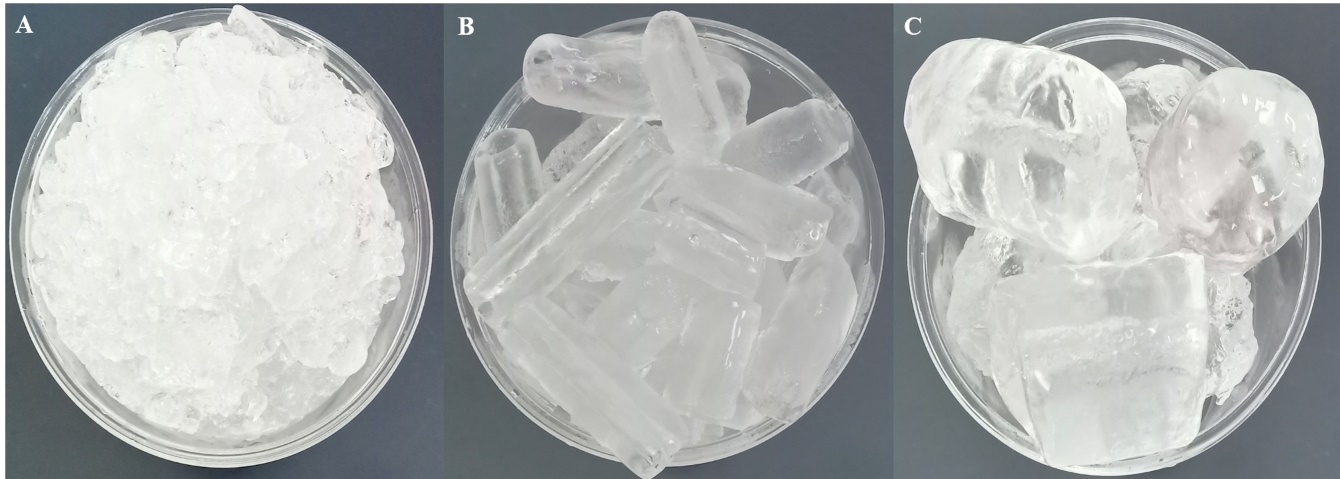


**Reference**

1. CLSI. Performance standards for antimicrobial susceptibility testing. *Clinical and Laboratory*

*Standards Institute: Wyne, PA.* 2021;31st Edition.
